# Family Caregiver Perspectives on Elder Mistreatment and Caring for Older Adults with Cognitive Impairment

**DOI:** 10.1093/geroni/igaf122.3140

**Published:** 2025-12-31

**Authors:** Karen Schlag, Leila Wood, Monique Pappadis

**Affiliations:** University of Texas Medical Branch at Galveston, Galveston, Texas, United States; University of Texas Health Science Center Houston, Houston, Texas, United States; University of Texas Medical Branch at Galveston, Galveston, Texas, United States

## Abstract

Older adults with cognitive impairment are at elevated risk for elder mistreatment (EM), with family caregiving strain recognized as a common risk factor. This qualitative study seeks to better understand how family caregiving for older adults with cognitive impairment and EM risk is influenced by interrelated interpersonal and social factors to provide a more holistic look at caregiving and vulnerability to mistreatment. Specifically, we examined perspectives from family caregivers about their knowledge of EM and their experiences with caregiving, carer strain, social support, and navigating healthcare systems. We conducted semi-structured interviews with 28 family caregivers from a culturally diverse, small urban U.S. community as part of a larger EM screening and support intervention study. Transcribed interviews were analyzed using reflexive thematic analysis. Drawing from a social-ecological framework, we identified three caregiver-focused themes: 1.) Tension between care recipient compliance gaining and elder mistreatment; 2.) Family dynamics and reduced patient care; 3.) Appropriate and desired healthcare interactions around EM. Findings underscore individual-, family-, and institutional-level influences on caregivers that can contribute to EM risk for older adults with cognitive impairment. Results also highlight a need for family-centered EM education and healthcare professionals’ proactive engagement with patients and families on caregiving stress management and patient safety. Finally, this research can inform future intervention efforts and policy to address EM of patients with cognitive impairment and their families.

